# Revealing the dance of NLRP3: spatiotemporal patterns in inflammasome activation

**DOI:** 10.1097/IN9.0000000000000053

**Published:** 2025-01-10

**Authors:** Lauren Spector, Naeha Subramanian

**Affiliations:** 1 Institute for Systems Biology, Seattle, WA, USA; 2 Department of Immunology, University of Washington, Seattle, WA, USA

**Keywords:** inflammasome, NLRP3, subcellular, organelle, innate immunity

## Abstract

The nucleotide-binding domain, leucine-rich repeat, and pyrin domain containing-protein 3 (NLRP3) inflammasome is a multiprotein complex that plays a critical role in the innate immune response to both infections and sterile stressors. Dysregulated NLRP3 activation has been implicated in a variety of autoimmune and inflammatory diseases, including cryopyrin-associated periodic fever syndromes, diabetes, atherosclerosis, Alzheimer’s disease, inflammatory bowel disease, and cancer. Consequently, fine-tuning NLRP3 activity holds significant therapeutic potential. Studies have implicated several organelles, including mitochondria, lysosomes, the endoplasmic reticulum (ER), the Golgi apparatus, endosomes, and the centrosome, in NLRP3 localization and inflammasome assembly. However, reports of conflict and many factors regulating interactions between NLRP3 and subcellular organelles remain unknown. This review synthesizes the current understanding of NLRP3 spatiotemporal dynamics, focusing on recent literature that elucidates the roles of subcellular localization and organelle stress in NLRP3 signaling and its crosstalk with other innate immune pathways converging at these organelles.

## 1. Introduction

The rapid detection of disruptions in tissue homeostasis and subsequent initiation of a context-dependent response is an essential function of the innate immune system. Germline-encoded pattern-recognition receptors (PRRs) accomplish this by sensing pathogen-associated molecular patterns and danger-associated molecular patterns which alert the immune system to infection or cellular damage ^[1]^. Signals from activated PRRs then launch an inflammatory response aimed at host defense or restoring homeostasis. Among these, the inflammasome-forming sensor proteins are cytosolic PRRs that trigger the release of inflammatory cytokines and cell death when activated.

NLRP3, a member of the nod-like receptor (NLR) family of cytosolic PRRs, forms an inflammasome that is one of the most extensively characterized, with its dysregulation being implicated in many autoinflammatory, autoimmune, and degenerative diseases ^[2–4]^. Gain-of-function mutations that render NLRP3 constitutively active cause the inflammatory symptoms characteristic of cryopyrin-associated periodic fever syndromes, a group of diseases encompassing familial cold autoinflammatory syndrome, Muckle-Wells syndrome, and neonatal-onset multisystem inflammatory disease ^[5–9]^. In addition to its prominent role in several autoimmune and autoinflammatory conditions, NLRP3 inflammasome activation has also been implicated in the pathology of both chronic and acute cardiovascular and metabolic diseases ^[6–9]^. Conversely, the impact of NLRP3 activation in cancer is context-dependent, and both pro-tumorigenic and antitumorigenic effects have been observed ^[8,10,11]^. Due to its role in modulating inflammation across a broad array of diseases, the NLRP3 inflammasome pathway has emerged as an attractive therapeutic target.

Canonical NLRP3 activation requires both “priming” and “activating” signals ^[3]^. Priming through toll-like receptor (TLR), tumor necrosis factor receptor (TNFR), interleukin-1 receptor (IL-1R) or nucleotide-binding oligomerization domain-containing protein 2 (NOD2) mediated signaling activates the transcription factor nuclear factor-kappa B (NF-кB) to upregulate expression of NLRP3 itself alongside pro-IL-1β and pro-IL-18. This priming step is also pivotal for post-translational modifications (PTMs) of NLRP3 that further regulate its readiness for activation ^[12]^. Several activating signals have been identified for NLRP3, ranging from alum, silica, and cholesterol crystals to adenosine triphosphate (ATP), as well as various bacterial, viral, and fungal pathogens ^[4]^. The proposed unifying mechanisms for such broad reactivity include the sensing of cellular alterations like K^+^ efflux, Ca^2+^ flux, mitochondrial dysfunction, lysosomal rupture, and endoplasmic reticulum (ER) stress—all pointing towards an intricate interplay with subcellular organelles that compartmentalize crucial yet distinct homeostatic cellular processes and highlighting how organelle dysfunction may manifest in diseases linked with aberrant NLRP3 activity ^[13]^. During activation, oligomerized NLRP3 recruits the adaptor protein apoptosis-associated speck-like protein containing a CARD domain (ASC) through pyrin domain (PYD-PYD) interactions, leading to the aggregation of ASC into a speck, followed by recruitment of pro-caspase-1 via caspase activation and recruitment domain (CARD-CARD) interactions ^[2–4]^. The assembled inflammasome activates caspase-1, which processes pro-IL-1β and pro-IL-18 to their active forms and cleaves gasdermin D to form membrane pores, thereby facilitating the release of bioactive IL-1β and IL-18 and leading to pyroptosis.

**Figure 1.**
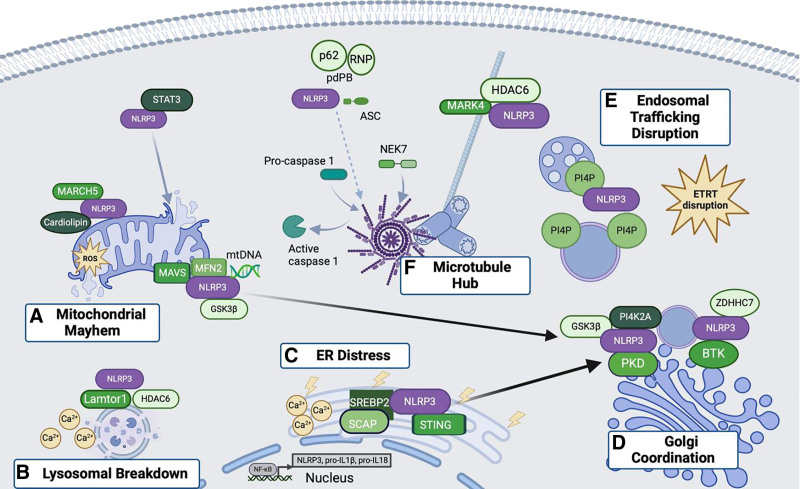
**Spatiotemporal regulation of NLRP3 inflammasome activation**. (A) Mitochondrial mayhem: Mitochondrial damage leads to ROS production and mtDNA release. GSK3β and phosphorylated STAT3 transport NLRP3 to the mitochondria. NLRP3 binds mtDNA leading to its activation. Interactions with MAVS, MFN2, and cardiolipin at the mitochondria promote inflammasome assembly. MARCH 5 deubiquitinates NLRP3, facilitating its interaction with NEK7. (B) Lysosomal breakdown: lysosome rupture induced by particulate activators leads to calcium flux and mitochondrial damage, activating NLRP3. NLRP3 associates with Lamtor1 and HDAC6, and HDAC6 enhances the NLRP3-Lamtor1 interaction, promoting inflammasome activation. (C) Endoplasmic reticulum (ER) distress: ER stress induces Ca^2+^ release and mitochondrial dysfunction, activating NLRP3. STING recruits and deubiquitinates NLRP3 at the ER, promoting activation. NLRP3 forms a complex with SCAP and SREBP2 for translocation to the Golgi, facilitating inflammasome assembly. (D) Golgi coordination: GSK3β phosphorylates PI4K2A to recruit NLRP3 from the mitochondria to the TGN. Additionally, the formation of a ternary complex with SREBP2 and SCAP facilitates NLRP3 translocation from the ER to a Golgi-mitochondrial interface. At the TGN, NLRP3 is phosphorylated by PKD. Additional post-translational modifications, including phosphorylation by BTK and palmitoylation by ZDHHC7, further enhance TGN localization and promote inflammasome assembly. (E) Endosomal trafficking disruption: disruptions in endosome trafficking, particularly in the endosome-to-TGN retrograde transport (ETRT), lead to PI4P accumulation on endosomal vesicles, recruiting NLRP3. (F) Microtubule hub: ASC and NEK7 recruitment, and full inflammasome assembly, occur at the MTOC or in the cytosol. MARK4 and HDAC6 promote the microtubule transport of NLRP3 to the MTOC. Additionally, a subset of the p62 protein phase separates with RNPs to form pd-PBs, recruiting ASC and NLRP3 and forming a scaffold for inflammasome assembly. Subcellular localization of pd-PBs in relation to the MTOC or other organelles is not known and is therefore indicated with a dashed line. NLRP3 interaction partners promoting inflammasome assembly are depicted in green. Proximity of green elements indicates known individual interactions with NLRP3, shown together for diagrammatic purposes; it does not imply simultaneous complex formation. Stimuli are shown in yellow. Solid black arrows represent an inter-organelle movement of NLRP3, and gray arrows indicate the translocation of NLRP3 and other inflammasome components from the cytosol to specific organelles, facilitated by the indicated proteins. Only direct interaction partners of NLRP3 are illustrated. ASC, apoptosis-associated speck-like protein containing a CARD domain; GSK3β, glycogen synthase kinase 3β; HDAC6, histone deactylase 6; MARCH 5, membrane-associated ring-CH-type finger 5; MARK4, microtubule-affinity regulating kinase 4; MAVS, mitochondrial antiviral signaling; MFN2, mitofusin 2; MTOC, microtubule organizing center; NEK7, NIMA-related kinase 7; NLRP3, nucleotide-binding domain, leucine-rich repeat, and pyrin domain containing-protein 3; PI4P, phosphatidylinositol-4-phosphate; Pd-PBs, p62-dependent P-bodies; PKD, protein kinase D; RNP, ribonucleoprotein; STAT3, Signal Transducer and Activator of Transcription-3; STING, stimulator of interferon genes; TGN, trans-Golgi network. Created in BioRender. Spector, L. (2024) https://BioRender.com/s17q872.

Studies have implicated multiple organelles in the localization and assembly of the NLRP3 inflammasome, yet many of these reports conflict, and the contributions of different organelles to activation remain unresolved. At rest, NLRP3 is localized to the cytosol ^[14–17]^ and the ER ^[18]^, as well as the nucleus ^[19,20]^ in some cell types. However, recruitment to various subcellular compartments has been found to promote inflammasome assembly. Upon activation, different reports demonstrate NLRP3 inflammasome formation at the mitochondria ^[15–17]^, mitochondria-associated ER membranes ^[14,18,21]^, lysosome membranes ^[22,23]^, Golgi apparatus ^[24–26]^, endosomes ^[22,27]^, and the centrosome ^[28,29]^ (Figure 1 and Table 1). Several prior reviews propose that NLRP3 is a sensor of organelle stress and is thus recruited to dysfunctional organelles ^[13,37–39]^. We review recent literature on the subcellular localization of the NLRP3 inflammasome and the roles of organelles and organelle stress in NLRP3 signaling. We discuss its crosstalk with other innate pathways converging at similar organelles whose concerted action is synthesized into an immune response and suggest that nonlinear, context-dependent behaviors exist within the spatiotemporal interactions that underlie NLRP3 activation.

**Table 1:** An overview of NLRP3 localization and activation, summarizing studies directly assessing NLRP3 subcellular location by imaging or subcellular fractionation methods.

NLRP3 localization (activated)	NLRP3 localization (at rest)	Cell type	Activator(s)	Methods	Reference
MAMs	ER	THP-1	MSU, rotenone, antimycin	Subcellular fractionation, confocal and electron microscopy	Zhou et al, 2011 ^[18]^
Mitochondria	Cytosol	HEK293T, BMDMs	Nigericin, ATP	Confocal microscopy, subcellular fractionation	Subramanian et al, 2013 ^[16]^
Mitochondria	Cytosol	J774A.1	Silica, linezolid	Subcellular fractionation	Iyer et al, 2013 ^[15]^
Mitochondria	Cytosol	BMDMs	ATP	Subcellular fractionation, immunofluorescence microscopy	Yang et al, 2015 ^[17]^
Golgi-adjacent MAMs then cytosol	Cytosol	THP-1, BMDMs	Nigericin, ATP	Confocal microscopy	Zhang et al, 2017 ^[14]^
Microtubules to mitochondria then MTOC	Cytosol	THP-1	Nigericin	Confocal microscopy	Li et al, 2017 ^[29]^
TGN	Cytosol and TGN	HeLa, BMDMs	Nigericin, ATP, imiquimod, CL097	Subcellular fractionation, immunofluorescence microscopy	Chen & Chen, 2018 ^[24]^
Mitochondria and MAMs	Cytosol	J774A.1	LPS, PamCSK4, or poly I:C priming alone and with nigericin	Subcellular fractionation	Elliot et al, 2018 ^[21]^
Mitochondria and Golgi	Cytosol	BMDMs, THP-1	ATP, nigericin	Subcellular fractionation, super resolution, and 3D structured illumination microscopy	Guo et al, 2018 ^[30]^
ER	Cytosol	HeLa, TPA-differentiated THP-1 macrophages	HSV-1 infection	Subcellular fractionation, confocal microscopy	Wang et al, 2020 ^[31]^
TGN then MTOC	Cytosol	iBMDMs, THP-1	Nigericin, MSU	Confocal microscopy	Magupalli et al, 2020^[28]^
Membrane-bound, TGN	Cytosol and membrane-bound	HEK293T, iBMDMs	Nigericin	Subcellular fractionation, confocal microscopy	Andreeva et al, 2021^[32]^
TGN	Golgi, cytosol	BMDMs	Nigericin	Subcellular fractionation, confocal microscopy	Bittner et al, 2021 ^[25]^
TGN	Cytosol	BMDMs, iBMDMs	Nigericin	Confocal microscopy	Nanda et al, 2021 ^[26]^
Mitochondria then TGN	Cytosol	HeLa, BMDMs	Nigericin, ATP	Confocal microscopy	Arumugam et al, 2022 ^[33]^
Endosomes and lysosomes	Cytosol and TGN	COS7	Nigericin, monensin	Immunofluorescence microscopy	Lee et al, 2023 ^[22]^
Endosomes	Cytosol	HeLa, THP-1	Nigericin, CL907	Confocal microscopy	Zhang et al, 2023 ^[27]^
Mitochondria	Cytosol	BMDMs	ATP, Hexokinase-trans-activator of transcription (HK-TAT)	Subcellular fractionation	Baik et al, 2023^[34]^
Mitochondria and MAMs	Cytosol	Primary human macrophages	Nigericin	Subcellular fractionation	Sharma et al, 2024 ^[35]^
Mitochondria	Cytosol	Mouse peritoneal macrophages	Nigericin, ATP	Immunofluorescence microscopy	Luo et al, 2024 ^[36]^

ATP, adenosine triphosphate; BMDM, bone marrow-derived macrophages, MAM, mitochondria-associated membranes; MSU, monosodium urate; MTOC, microtubule organizing center; NLRP3, nucleotide-binding domain, leucine-rich repeat, and pyrin domain containing-protein 3; TGN, trans-Golgi network.

## 2. Mitochondria

Previous studies suggest that NLRP3 senses markers of mitochondrial dysfunction including reactive oxygen species (ROS) and mtDNA release ^[18,40,41]^. Common NLRP3 agonists such as nigericin cause mitochondrial damage and increase mtDNA release, ROS production, and oxidization of mtDNA. Binding to oxidized (ox) mtDNA can directly activate the NLRP3 inflammasome ^[41,42]^. Unlike ox-mtDNA, nonoxidized mtDNA was shown to activate AIM2, not NLRP3. However, Cabral et al ^[43]^ recently demonstrated that wild-type NLRP3 can bind both ox and nonox mtDNA, with a greater affinity for unoxidized mtDNA. On the other hand, a mutant of NLRP3 that causes NOMID showed greater affinity for ox-mtDNA, suggesting that the binding interaction with ox-mtDNA regulates the sensitivity of NLRP3 to activators ^[43]^. Whether interaction with nonox-mtDNA plays a role in inflammasome activation is yet to be determined.

Aside from direct binding with ox-mtDNA, other factors linked to the mitochondria including mitochondrial enzymes, DNA synthesis, and metabolism have also been implicated in NLRP3 activation. The dissociation of hexokinase, an enzyme that senses bacterial peptidoglycan, from the mitochondrial outer membrane is sufficient for NLRP3 activation ^[34]^. MtDNA synthesis driven by a priming signal like LPS is also required, and inhibiting mtDNA replication through ablation of transcription factor A mitochondrial and mtDNA polymerase prevents NLRP3 activation ^[44]^. However, Billingham et al ^[45]^ later found that ATP generation by a functional electron transport chain (ETC) is necessary for NLRP3 activation ^[45]^. Therefore, impaired mtDNA replication may inhibit NLRP3 activation through downstream effects on the ETC. In the same study, Billingham and colleagues reconstituted bone marrow-derived macrophages (BMDMs) lacking functional ETC complex III with *Ciona intestinalis* alternative oxidase (AOX) which allows for normal electron flow and ATP generation but not ROS production. These cells activated NLRP3 to the same degree as WT BMDMs. Further, mice conditionally expressing AOX in myeloid cells lacking mitochondrial complex III function produced a similar amount of IL-1β to WT mice upon LPS challenge. These new data, consistent with older studies, suggest that while ROS is associated with NLRP3 activators, it may not cause NLRP3 activation.

Mitophagy negatively regulates NLRP3 activation by multiple mechanisms, most commonly by limiting mtDNA and mtROS release from damaged mitochondria ^[46–48]^. Early work showed that BMDMs lacking key autophagy genes accumulate damaged mitochondria, leading to an increase in NLRP3 activation ^[49]^. A 2021 study subsequently implicated an early endosome-dependent mitophagy pathway in repressing NLRP3 activation in macrophages ^[48]^. Several studies point to the selective autophagy receptor, p62, as a crucial regulator that links autophagy, mitophagy, and NLRP3 inflammasome activation. Early work showed that recruitment of p62 to ubiquitinated, assembled inflammasomes leads to their degradation in autophagosomes, limiting IL-1β production ^[50]^. Subsequently, Zhong et al ^[46]^ found that NF-кB signaling upregulates the expression of p62, which is then translocated to damaged mitochondria inducing mitophagy to reduce NLRP3 inflammasome activation and assembly. Consistent with these findings, the transcription factor MafB was found to increase p62 expression and inhibit mitochondrial damage, ROS production, and mtDNA release, thereby inhibiting NLRP3 inflammasome activation ^[51]^. MafB deficiency increased NLRP3 activation in vitro and in vivo. Thus, NF-кB signaling may form a negative feedback loop via p62 to constrain the NLRP3 inflammasome after activation. However, recent work from Barrow and colleagues ^[52]^ supports a more complex role for p62 in NLRP3 activation ^[52]^. They found that a subset of the p62 protein phase separates with ribonucleoproteins in response to LPS priming and nigericin treatment, forming unique p62-dependent P-bodies that recruit ASC to provide a platform for rapid inflammasome assembly. This suggests an intriguing ability for p62 to both negatively and positively regulate the NLRP3 inflammasome. The factors balancing these potentially opposing roles of p62 in NLRP3 activation are still unknown. Overall, the role of mitophagy and autophagy proteins in regulating NLRP3 at various stages of activation remains an exciting avenue of research.

Several studies have found that NLRP3 associates with the mitochondria during activation and that this localization is required for optimal function. Mitochondrial antiviral signaling (MAVS) protein interacts directly with the NLRP3 *N*-terminus to recruit NLRP3 to the mitochondrial surface to promote inflammasome assembly ^[16,53–55]^. This interaction between MAVS and NLRP3 may also function to inhibit type I interferon signaling in favor of inflammasome activation ^[53]^. Additional interaction partners promoting NLRP3 activation and mitochondrial localization include mitofusin 2 ^[56]^, which was found to strengthen the NLRP3-MAVS interaction in response to RNA virus infection, and mitochondrial cardiolipin ^[15]^, found to directly bind and recruit NLRP3 to dysfunctional mitochondria. Later work demonstrated that cardiolipin binds caspase-1 in response to LPS, supporting its role as a scaffold for inflammasome activation ^[21]^. This process may be negatively regulated by competitive binding between cardiolipin and cytochrome c, promoting apoptosis rather than pyroptosis ^[57]^. Another study found that the mitochondria-associated E3 ligase membrane-associated ring-CH-type finger 5 (MARCH 5) ubiquitinates NLRP3 at the mitochondrial membrane, licensing its subsequent interaction with NIMA-related kinase 7 (NEK7) for optimal oligomerization and inflammasome assembly ^[58]^. Finally, recent work found that signal transducer and activator of transcription-3 (STAT3) not only promotes inflammasome activation by increasing NLRP3 expression ^[59,60]^ and preventing mitophagy ^[61]^ but also transports NLRP3 from the cytosol to the mitochondria ^[36]^. These data collectively point to the mitochondrial membrane serving as a critical scaffolding site for the NLRP3 response to mitochondrial damage.

## 3. Endoplasmic reticulum and mitochondria-associated membranes

Like mitochondria, the endoplasmic reticulum interacts with the NLRP3 inflammasome in several ways: it activates NLRP3 due to ER stress ^[62–66]^, engages in a complex system of ER-mitochondrial crosstalk ^[35,67,68]^, regulates intracellular cholesterol levels ^[30,69]^, and serves as an additional site for NLRP3 localization ^[14,18,21,31]^. While several reports position resting NLRP3 in the cytosol, Zhou et al ^[18]^ found that NLRP3 predominantly localizes to the ER in unstimulated THP-1 cells, and colocalizes with ASC at ER-mitochondria-associated membranes (MAMs) in the perinuclear space upon activation, rather than at the membranes of free mitochondria ^[18]^.

ER stress can cause NLRP3 activation through multiple mechanisms. It has been demonstrated that ER stress can trigger mitochondrial dysfunction independent of the classical unfolded protein response, resulting in subsequent activation of NLRP3 ^[65]^. Furthermore, Ca^2+^ release from the ER has been shown to activate NLRP3 in BMDMs, and this mechanism plays a critical role in the aberrant NLRP3 activation observed in cryopyrin-associated periodic syndromes patients ^[70]^. Given that MAMs facilitate Ca^2+^ transfer between the ER and mitochondria ^[71]^, this also supports a role for MAMs as a site for inflammasome activation. Additionally, crosstalk at the MAM interface finetunes the NLRP3 response to mitochondrial damage, and the orphan nuclear receptor small heterodimer partner has been identified as a regulator of this process in macrophages ^[17]^.

Several reports also link the ER-resident sensor inositol-requiring enzyme 1 (IRE1) to ER stress-induced inflammasome activation ^[62,64,66,72]^. Activated IRE1 upregulates thioredoxin-interacting protein (TXNIP), which associates with thioredoxin-2 at the mitochondria to promote ROS release ^[66]^. This prompts NLRP3 and caspase-2 recruitment to the mitochondria, ultimately leading to loss of mitochondrial membrane integrity and inflammasome assembly ^[62]^. Downstream of IRE1, downregulation of X-box binding protein 1 (XBP1) has been shown to reduce NLRP3 activation in a kidney injury model ^[68]^. Further investigations into ER-mitochondrial crosstalk at MAMs have confirmed that ER stress in monocytes leads to NLRP3 activation via Ca^2+^ transfer from the ER to mitochondria ^[67,70]^. This is associated with closer ER-mitochondrial junctions and mitochondrial depolarization ^[67]^. Likewise, recent findings have identified a role for orosomucoid-like protein 3 in enabling mitochondrial fragmentation and closer ER-mitochondrial contacts to promote NLRP3 activation in an ulcerative colitis model ^[35]^.

A primary function of the ER is to maintain lipid homeostasis, and the presence of cholesterol in the ER is required for NLRP3 recruitment to MAMs and ASC in macrophages ^[69]^. The precise mechanism behind how ER cholesterol promotes inflammasome assembly remains unknown. Additional work implicates stimulator of interferon genes (STING) in regulating NLRP3 inflammasome assembly at the ER. In response to herpes simplex virus type 1 (HSV-1) infection, STING recruits NLRP3 to the ER, facilitating its deubiquitination to promote inflammasome assembly ^[31]^. This crosstalk between NLRP3 and cGAS-STING at the ER is crucial for host defense against HSV-1.

## 4. Lysosomes and phagolysosomes

Lysosome destabilization has long been implicated in NLRP3 activation. Phagocytosis of particulate activators including silica, cholesterol crystals, calcium pyrophosphate crystals, and amyloid-β aggregates leads to K+ efflux via lysosome rupture ^[73–77]^. Because cathepsin inhibitors prevent NLRP3 activation by particulates, cathepsin release from lysosomes into the cytosol has been hypothesized to trigger inflammasome activation ^[78–81]^. However, cathepsin knockout BMDMs still exhibit NLRP3 activation in response to lysosomal disruptors, and treating these cells with cathepsin inhibitors still inhibits NLRP3 activation, suggesting that additional targets of these inhibitors may be involved ^[81]^.

In 2017, Gaidt et al ^[82]^ found that cGAS-STING activation in human monocytes can also lead to lysosome disruption, K+ efflux, and NLRP3 activation. This aligns with recent work showing that activating the cGAS-STING axis causes mtDNA escape, likewise activating NLRP3 ^[41]^. The interplay between lysosome disruption, mitochondrial damage, and NLRP3 activation is further supported by a 2023 study identifying apilimod as a specific NLRP3 activator that induces calcium release from lysosomes, leading to mitochondrial damage and ROS production ^[83]^. Similarly, the Mitochondrial Ca^2+^ Uniporter promotes silica and alum-induced NLRP3 activation in macrophages by inhibiting phagolysosome membrane repair ^[84]^, illuminating the connection between mitochondrial Ca^2+^ signaling, phagolysosome integrity, and NLRP3 activation.

Finally, it was discovered that Lamtor1, a component of the lysosomal membrane Ragulator complex, interacts with both NLRP3 and histone deacetylase 6 (HDAC6) resulting in inflammasome activation in both murine macrophages and THP-1 cells and ASC speck formation near the lysosome ^[23]^. This suggests that not only does loss of lysosome integrity activate NLRP3, but the lysosome membrane may also serve as an additional hub for inflammasome assembly.

## 5. Golgi/trans-Golgi network

The Golgi apparatus and the trans-Golgi network (TGN) are essential components of the cell’s secretory pathway, responsible for processing, modifying, and sorting proteins and lipids for transport to their final destinations. The implication of the Golgi apparatus in NLRP3 activation began to emerge in 2017 ^[14]^. Since then, several studies have highlighted the role of the Golgi and TGN in NLRP3 activation. Initially, Zhang et al ^[14]^ observed increased diacylglycerol levels (DAG) at the Golgi after treatment with NLRP3 activators, correlating with NLRP3 localization at Golgi-adjacent MAMs. DAG recruited protein kinase D to phosphorylate self-oligomerized NLRP3, allowing its release from MAMs to assemble into an inflammasome in the cytosol. This and a later study also found that disrupting ER-Golgi trafficking with brefeldin A inhibits NLRP3 inflammasome assembly ^[85]^.

Further support for the involvement of Golgi in NLRP3 activation came in 2018 from a study noting the recruitment of NLRP3 to the TGN through binding with phosphatidylinositol-4-phosphate (PI4P) at the Golgi membrane ^[24]^. The authors demonstrated that the TGN forms dispersed vesicles in the perinuclear space upon nigericin treatment, colocalizing with overexpressed NLRP3 in HeLa cells and in ASC^−/−^ BMDMs. However, it remained unclear whether full inflammasome assembly, including ASC speck formation, occurs at the TGN or another site. Surprisingly, this study did not observe NLRP3 localization at the mitochondria, instead proposing the TGN as the key scaffolding site for inflammasome activation. Around the same time, it was reported that NLRP3 translocation from the ER to the Golgi in mouse macrophages and THP-1 cells upon activation requires association with a complex comprised of sterolregulatory element-binding protein 2 (SREBP2) and SREBP cleavage activating protein (SCAP), a regulator of cholesterol metabolism ^[30]^. This study, however, found NLRP3 in both mitochondrial and Golgi subcellular fractions while associated with SCAP.

Arumugam and colleagues ^[33]^ sought to resolve discrepancies in prior reports through live tracking of NLRP3 localization. They proposed a model whereby NLRP3 initially colocalizes with mitochondria within 10–15 minutes postactivation before translocating to the TGN. The authors also found that glycogen synthase kinase 3β facilitates initial NLRP3 recruitment to the mitochondria through direct binding, and later promotes its oligomerization at the TGN through phosphorylation of phosphatidylinositol-4-kinase 2 Α promoting inflammasome assembly.

Other studies have identified Bruton’s tyrosine kinase (BTK) ^[25]^ and inhibitor of nuclear factor kappa-B kinase subunit beta (IKKβ) ^[26]^ as regulators of NLRP3 recruitment to the TGN. BTK directly phosphorylates NLRP3, while the phosphorylation target of IKKβ remains unknown.

Additional PTMs of NLRP3 regulating its localization to the TGN have recently been uncovered. *S*-palmitoylation of NLRP3 Cys126 by zinc finger DHHC-type palmitoyl transferase 7 (ZDHHC7) facilitates localization of resting and nigericin-activated NLRP3 to the TGN and palmitoylation is critical for ASC recruitment ^[86]^. In contrast, NLRP3 palmitoylation at Cys844 by ZDHHC12 at the TGN leads to lysosomal degradation of NLRP3 via the chaperone-mediated autophagy pathway ^[87]^. Finally, the *S*-acylation of NLRP3 Cys130 by ZDHHC enzymes has also been implicated in its recruitment to and immobilization at the Golgi after nigericin activation ^[88]^. This may likewise impact NLRP3 oligomerization, although the precise mechanism of how this modification impacts activation and inflammasome assembly is not yet known. Overall, PTMs at distinct sites of NLRP3 by ZDHHC enzymes appear to sequentially regulate NLRP3 activation and inhibition ^[86–89]^.

## 6. Endosomal network

Recent studies have highlighted the significant role of the endosomal network in NLRP3 activation and TGN vesicle dispersal ^[22,27]^. It has been proposed that NLRP3 senses defective endosome trafficking, which is important for its activation. NLRP3 activators, including nigericin, imiquimod, and LeuLeu-OMe, disrupt endosome trafficking pathways, and NLRP3 localizes to endosomal vesicles and the lipid PI4P ^[22]^. However, while treatment with monensin, a chemical disruptor of endosomal trafficking, potentiated NLRP3 inflammasome activation, it was not sufficient to induce activation in LPS-primed BMDMs without another stimulus. In a complementary study, Zhang et al ^[27]^ reported that NLRP3 activators disrupt endosome trafficking and increase the endosomal accumulation of PI4P, which recruits NLRP3 to endosomes and triggers inflammasome activation.

The role of PI4P in this process is particularly intriguing. Typically associated with the Golgi apparatus, PI4P can accumulate on endosomes due to disruptions in endoplasmic reticulum–endosome membrane contact sites and endosome-to-trans-Golgi network retrograde trafficking (ETRT) ^[27]^. These disruptions increase in endosomal PI4P levels, facilitating NLRP3 recruitment to endosomes. Reducing endosomal PI4P levels prevents NLRP3 association with endosomes and inhibits inflammasome activation ^[22,27]^. Additionally, TGN38 and its human homolog TGN46, which are commonly used markers of the TGN, cycle between the plasma membrane and TGN through endosomes ^[90]^. Treatment with NLRP3-activating stimuli or monensin impairs this cycling from early endosomes to the TGN, causing an accumulation of TGN38/46 in early endosomes ^[22]^. These findings align with a prior report that during inflammasome activation, NLRP3-positive vesicles contain both the early endosome marker EEA1 and TGN marker TGN38 ^[24]^. However, they support a revised model in which NLRP3-positive vesicles originate from the endosomes rather than the Golgi, with TGN46 enriched on endosomes due to a defect in ETRT. Given these results, it will be important to clearly distinguish endosomes from the TGN in future studies of NLRP3 localization. For instance, it is possible that rather than proceeding from the mitochondria to the TGN during activation as suggested by Arumugam et al ^[33]^, NLRP3 first translocates from the mitochondria to endosomes. Still, these new studies provide a clearer understanding of the spatial dynamics of NLRP3 activation, linking the endosomal network, TGN, and the accumulation of PI4P on endosomes in this process.

## 7. Centrosome/microtubule organizing center

While many studies demonstrate localization of NLRP3 at membrane-bound organelles during inflammasome activation, these membrane-associated NLRP3 speckles are not all colocalized with a single ASC speck characteristic of the fully formed inflammasome. Emerging evidence suggests that the final assembly of the NLRP3 inflammasome occurs at the centrosome, with localization to other organelles serving as intermediary steps. Prior work has shown that microtubules are essential for NLRP3 activation ^[29,91]^. Microtubules spatially rearrange damaged mitochondria to activate NLRP3 at the ER ^[91]^. Moreover, the centrosome protein NEK7 was found to interact with NLRP3 and promote its oligomerization in response to potassium efflux ^[92,93]^. In 2017, Li et al ^[29]^ showed that microtubule-affinity regulating kinase 4 (MARK4) transports activated NLRP3 to the mitochondria and subsequently to the microtubule organizing center (MTOC) where ASC speck formation occurs. This model was supported by work from Magupalli et al ^[28]^ in 2020 which showed that the dynein adaptor HDAC6 is required for microtubule transport of NLRP3 and inflammasome assembly at the MTOC. The authors also found that NEK7 fails to colocalize with NLRP3 at the TGN before HDAC6-mediated microtubule transport. Instead, NEK7 engages NLRP3 only after it is delivered by HDAC6 to the MTOC (where NEK7 resides) for final inflammasome assembly. Such assembly and activation of NLRP3 at the centrosome is modulated by the centrosomal protein Spata2 which negatively regulates the NEK7-NLRP3 interaction, suppressing inflammasome activity ^[94]^. Mechanistically, Spata2 recruits the deubiquitinase cylindromatosis lysine 63 deubiquitinase (CYLD) to promote deubiquitination of polo-like kinase 4 (PLK4), a regulator of centrosome duplication. Deubiquitinated PLK4 then binds and phosphorylates NEK7 to inhibit its interaction with NLRP3 at the centrosome. Together, these data suggest that full inflammasome assembly occurs at the MTOC, not at the TGN and that centrosome-associated regulatory mechanisms finely tune this process to ensure precise inflammasome activation.

Mechanisms of NLRP3 regulation at the centrosome following inflammasome assembly have also been identified. Yang and colleagues ^[94]^ found that NLRP3 inflammasome localization to the centrosome is transient in LPS/nigericin-treated BMDMs, and the fully functional inflammasome may then be released into the cytosol. This may allow for its subsequent release into the extracellular space to amplify inflammatory signaling ^[95]^. Alternatively, the assembled inflammasome can be degraded in autophagolysosomes to resolve signaling. Assembled NLRP3 inflammasomes are enriched in LC3b, an RNA-binding protein critical for mRNA degradation during autophagy, and surrounded by an interrupted double membrane, indicating autophagy induction ^[28]^. Despite these insights, the precise mechanisms that terminate inflammasome activation remain largely unexplored. The regulatory factors determining the fate of assembled NLRP3 inflammasomes (degradation or extracellular release) have yet to be determined, highlighting an important area for future research.

Translocation of NLRP3 to the MTOC may not be essential for inflammasome function in all cases. A detailed structural study of endogenous ASC specks in THP-1 cells following NLRP3 activation with nigericin found that ASC specks do not colocalize to the MTOC 90 minutes post-treatment ^[96]^. This later timepoint, compared to the earlier timepoints examined by Magupalli et al ^[28]^ and Yang et al ^[94]^, suggests that ASC specks either migrate to the cytosol after inflammasome assembly at the MTOC and/or that inflammasome assembly occurs freely in the cytosol in some cells. Recent studies have also highlighted the context-dependent nature of HDAC6 involvement in inflammasome assembly, showing that HDAC6 is required for optimal inflammasome assembly in certain cell types under specific conditions, but not universally required. Specifically, HDAC6 involvement was observed in immortalized BMDMs, and partially in THP-1 cells and human peripheral blood mononuclear cells but not primary mouse BMDMs ^[97]^. HDAC6 inhibition appears to suppress the NLRP3 inflammasome most efficiently at early timepoints and in response to monosodium urate rather than nigericin, indicating that the role of HDAC6, and by extension the MTOC, may vary depending on the cell type, timepoint, and activating stimulus. Overall, the evidence indicates that the centrosome may serve as the final signaling hub for NLRP3 inflammasome assembly in some contexts, although the precise mechanisms governing this process remain to be fully elucidated.

## 8. Concluding remarks

The literature supports a highly dynamic model of NLRP3 activation and localization in response to organelle stress (Figure 1 and Table 1), with several avenues for continued research. It is generally believed that resting NLRP3 exists in a monomeric state and that activation induces oligomerization. However, structural analysis has identified a specific double-ring 12-16-mer cage form of mouse NLRP3 as critical for TGN dispersion into vesicles and subsequent inflammasome activation ^[32]^. This NLRP3 oligomer is predominantly membrane-localized, suggesting a unique role as a sentinel for organelle dysfunction. A complementary paper reports cryo-electron microscopy structures of human NLRP3 forming a decamer cage in its inactive state and when complexed with the inhibitor CRID3 (also referred to as MCC950) ^[98]^. Further work is needed to identify distinctions in the oligomerization status and structure of NLRP3 at various localization sites and how this relates to function.

Post-translational modifications of NLRP3 during priming and activation including phosphorylation, ubiquitination, and acetylation have long been known to regulate inflammasome assembly, and several have also been shown to impact localization ^[3,12,14,25,58,86–89,99,100]^. The contributions of PTMs at different stages of NLRP3 activation to its translocation between organelles offer additional layers of regulation to unravel. Furthermore, the NLRP3 LRR undergoes alternative splicing, and several splice isoforms cannot assemble into a functional inflammasome ^[101,102]^. The contribution of these splice variants to NLRP3 localization within the cell is still unknown.

While precise control of NLRP3 subcellular localization is clearly important for inflammasome assembly, it is not certain whether all the organelles identified in the literature are required in all cell types or in response to all NLRP3 activators. Most research has been conducted in macrophages and monocytes, and it is unclear if NLRP3 displays different localization patterns in neutrophils or in endothelial, epithelial, T or B cells. Additionally, cell-to-cell heterogeneity plays a role in NLRP3 activation. Not every cell in a presumably homogeneous population forms an inflammasome, suggesting that stochastic factors may also be at play. NLRP3 inflammasome activation can thus be viewed as an emergent property of the cell state, influenced by organelle stress, PTMs, splice variants, and inherent cellular variability. Available treatments to inhibit the NLRP3 inflammasome pathway include IL-1 receptor antagonists and IL-1β antibodies such as anakinra and canakinumab ^[103,104]^. Due to their specificity, drugs directly inhibiting NLRP3 reduce the risk of opportunistic infection and tend to have better safety profiles. Several NLRP3 inhibitors including Dapansutrile (Olatec Therapeutics), DFV-890 (Novartis), NT-0796 (NodThera), ZYIL1 (Zydus Life Sciences), and VTX-2735 (Ventyx Biosciences) have reached Phase II of clinical trials ^[104]^. While many unanswered questions remain, recent discoveries regarding the spatiotemporal regulation of NLRP3 may translate into new potential treatments for disease. For example, the FDA-approved drug disulfiram inhibits NLRP3 palmitoylation, preventing its localization at the TGN ^[105–107]^. Ongoing work may repurpose disulfiram and other drugs as treatments for NLRP3-mediated inflammatory diseases. Additionally, STAT3 inhibitors such as napabucasin ^[36]^, the FDA-approved drug colchicine ^[60]^ and ODZ10117 ^[108]^ may be pursued to prevent NLRP3 translocation to the mitochondria. Continued investigation into the nuances of NLRP3 localization and the factors influencing its activation will enable a more comprehensive understanding of its role in disease, with myriad implications for therapeutic targeting.

## Conflicts of interest

The authors declare no conflict of interest.

## Funding

This work was supported by NIH grant R01AI163131 “NLRP3 inflammasome activation and its crosstalk with RLR signaling at the mitochondria” to NS. We apologize to all authors whose important contributions could not be included in this review due to space limitations.
